# A Charge of Professional Piracy

**Published:** 1849-07

**Authors:** 


*A Charge of Professional Piracy.*—Mr. Tomes whose series of valuable lectures we have been publishing in the Journal, has lately issued in England, the entire series, in a collected form, with some few additions, amongst which he refers to a practitioner, (Clendon,) who wrote a small work on the construction of forceps, three years after Mr. Tomes' papers with delineations had appeared in the *Medical Gazette*, and to which he never refers, although availing himself of the exact copy and description of the instruments. It unquestionably remains with Mr. Clendon to refute this charge and remove the stigma cast upon his professional reputation, before he can be justly entitled to rank amongst the scientific practitioners in Europe. The charge is publicly made—it must be as publicly refuted—we give the extract from the work.

"In 1841 I described, and figured with some pleasure, what I believed to be new and much improved forms of tooth-forceps, as possessing great advantages over the key instrument, then almost universally used. Now, after the lapse of seven years, I am again on the same subject, and I shall proceed with increased pleasure, for, during the interval, the instruments in question have come into general use. Nay, in further testimony of the value of the improved forceps, a member of my own profession, after ordering a set of my instrument-maker, found them, on trial, so serviceable, that he was induced to write a little work, minutely describing them, and strongly recommending their general adoption, to the exclusion of the instruments then in use, though, for the honor and good name of our profession, I regret to say he altogether neglected to acknowledge by whom the instruments, of which he had availed himself, and was describing, were designed and brought to their present state of utility, and published.

Indeed, so anxious has this author been to take to himself whatever credit there may be in the improvements, that he has allowed or induced many makers to stamp his name on each instrument."—p. 322. R.

